# Planar Cell Polarity Signaling in Collective Cell Movements During Morphogenesis and Disease

**DOI:** 10.2174/138920212803759721

**Published:** 2012-12

**Authors:** Verónica Muñoz-Soriano, Yaiza Belacortu, Nuria Paricio

**Affiliations:** Departamento de Genética, Facultad de CC Biológicas, Universidad de Valencia, Burjassot 46100, Valencia, Spain

**Keywords:** Collective cell movements, Development, *Drosophila*, Disease, Morphogenesis, Planar cell polarity.

## Abstract

Collective and directed cell movements are crucial for diverse developmental processes in the animal kingdom, but they are also involved in wound repair and disease. During these processes groups of cells are oriented within the tissue plane, which is referred to as planar cell polarity (PCP). This requires a tight regulation that is in part conducted by the PCP pathway. Although this pathway was initially characterized in flies*, *subsequent studies in vertebrates revealed a set of conserved core factors but also effector molecules and signal modulators, which build the fundamental PCP machinery. The PCP pathway in *Drosophila* regulates several developmental processes involving collective cell movements such as border cell migration during oogenesis, ommatidial rotation during eye development, and embryonic dorsal closure. During vertebrate embryogenesis, PCP signaling also controls collective and directed cell movements including convergent extension during gastrulation, neural tube closure, neural crest cell migration, or heart morphogenesis. Similarly, PCP signaling is linked to processes such as wound repair, and cancer invasion and metastasis in adults. As a consequence, disruption of PCP signaling leads to pathological conditions. In this review, we will summarize recent findings about the role of PCP signaling in collective cell movements in flies and vertebrates. In addition, we will focus on how studies in *Drosophila *have been relevant to our understanding of the PCP molecular machinery and will describe several developmental defects and human disorders in which PCP signaling is compromised. Therefore, new discoveries about the contribution of this pathway to collective cell movements could provide new potential diagnostic and therapeutic targets for these disorders.

## INTRODUCTION

Cellular polarization is essential for the function and development of many tissues. In epithelia, cells not only acquire apico-basal polarity but they are also polarized within the epithelial plane, which is known as planar cell polarity (PCP). Although initially described in epithelial tissues, PCP is also seen in non-epithelial cell types such as mesenchymal cells [1]. PCP establishment is required for organization of multicellular structures and tissue remodelling. However, it is also involved in the control of polarized cell migration and coordinated cell movements. Therefore, disruption of PCP can lead to developmental defects and disease (reviewed in [2, 3]).

**Fig. (1):**
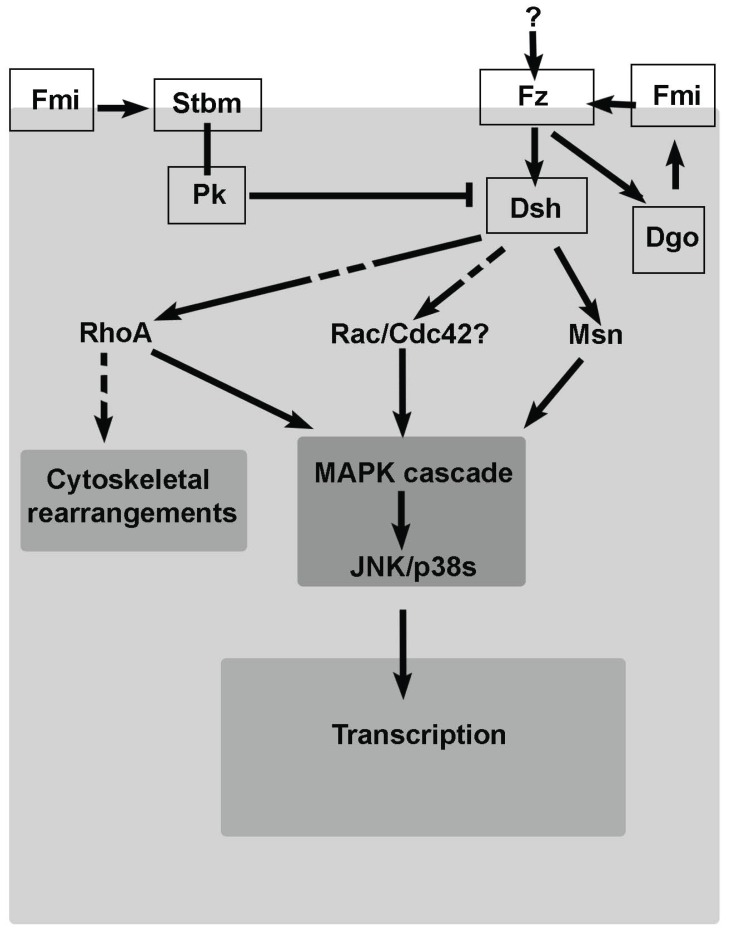
**The core PCP module in *Drosophila*.** Simplified schematic representation of the main components of the core PCP pathway in *Drosophila* as well as their relationships. The core PCP factors have been represented inside an outlined box.

PCP was initially discovered in *Drosophila* and other insects [4-6]. Indeed, the most thoroughly studied tissues in the context of PCP have been the fly eyes, wings, abdomen, and notum. PCP in these tissues is reflected by the perfect alignment of actin hairs on the wing and the abdomen, of bristles and sensory organs on the notum, and by the ordered array of ommatidia in the compound eye (reviewed in [7]). Genetic and molecular characterization of PCP mutants in this organism, in which disorganization of cuticular structures and/or compound eyes were observed, led to discover that PCP establishment requires a tight regulation that is in part uncovered by the Frizzled (Fz)/PCP signaling pathway (also known as non-canonical Wnt pathway). It has been proposed that the PCP signaling mechanism consists of two major groups of proteins: the core PCP module and the Fat/Dachsous (Ft/Ds) system (also called global module) (reviewed in [2, 7-10]). The core PCP genes are required to establish molecular asymmetry within and between cells, and encode transmembrane proteins as well as cytoplasmic components that associate with the membrane during PCP signaling. These proteins adopt polarized subcellular distribution, accumulating in proximal and distal subsets on opposite sides of cell-cell junctions. In flies, the transmembrane receptor Fz and the cytoplasmic proteins Dishevelled (Dsh) and Diego (Dgo) are localized in distal cell junctions while the transmembrane protein Strabismus (Stbm)/Vang Gogh (Vang) and the cytoplasmic protein Prickle (Pk) lie proximally. Besides, Flamingo (Fmi), another transmembrane protein, is localized both proximally and distally (Fig. **1**). The Ft/Ds system includes the atypical cadherins Ft and Ds, which interact heterophilically across membranes, and the Golgi protein Four-jointed (Fj) that modulates their affinity by phosphorylation. Ft and Ds display opposing expression gradients in polarized tissues that could provide directional information. However, it is still unknown whether the Ft/Ds module has a role upstream the core PCP module or whether it represents an independent and parallel system during PCP establishment [11]. In addition, many proteins that function as downstream effectors of PCP signaling have been described. Some of them function in all tissues analyzed such as the Rho family of GTPases or a JNK/p38 MAPK module (Fig. **1**), but most show tissue specificity and provide a link between this pathway and the cell-type specific responses required to generate PCP in each tissue. Interestingly, subsequent works in vertebrates have led to establish that PCP signaling is evolutionary conserved. Indeed, most of the PCP genes described in *Drosophila* have orthologs in vertebrates, in which multigene families of PCP proteins are required for proper tissue polarity (see below). However, Wnt ligands have been only described in vertebrates to regulate PCP and it remains still unknown how cells are initially polarized in *Drosophila*. Moreover, there is a certain mechanistic divergence between flies and vertebrates obviously due to the different morphological processes dependent on PCP establishment and the existence of aspects exclusive of vertebrates [10]. A detailed information about genes encoding PCP signaling components, regulators and effectors, both in *Drosophila* and vertebrates, can be found elsewhere [2, 7-10].

As mentioned above, the PCP pathway is active in polarized cells and tissues, but it is also required in several processes involving directional cell migration and collective cell movements [1, 9, 10, 12]. Cell migration is an essential and highly regulated process for countless developmental, homeostatic, and regenerative events in flies and vertebrates, but it also occurs under pathological conditions like cancer (see below). Although several cell types move during all their life and usually migrate alone, others do it only at specific developmental stages and in certain situations such as organogenesis or tissue repair, and often move in groups. This type of cell movement is referred to as collective cell movement (CCM), and can occur in tightly or loosely associated groups of cells (reviewed in [13-16]). A CCM is defined as a phenomenon in which cells not only move together but also make contact at least some of the time and they affect each other while moving or migrating [15]. Several forms of CCMs have been described but in all cases the molecular and cellular mechanisms underlying such movements imply cell-cell adhesion, collective cell polarization and changes in cytoskeletal activity, processing of directional cues, and position changes relative to the substrate (reviewed in [13-16]). One type of CCM is epithelial sheet migration, in which cells maintain close contact and continuity during the process. This type of movement takes place in tissue repair after wounding and in related developmental processes such as dorsal closure in *Drosophila* [17] and early morphogenetic movements in some animals [14-16]. Other cells move collectively in order to form elaborated cellular structures by sprouting and branching migration. While sprouting is characterized by the formation of a multicellular outgrowth from a unique tip cell, branching involves growth and shaping of large groups of cells into branched structures. Examples of such movements can be observed either during the formation of the *Drosophila* tracheal system or in mammary gland development, respectively [14-16]. Another possibility is that cells move collectively but in a loose arrangement, a process known as cell streaming or chain migration. The migration of neural crest (NC) cells is an example of this type of CCM. The NC is an embryonic population of cells that are specified in the neural tube from which they detach upon induction. Subsequently, they migrate and colonize almost the entire embryo, and differentiate to form diverse cell types [18]. Finally, several cells migrate or move in small clusters as free groups, in which they are tightly associated. Border cell migration in the ovary and rotation of photoreceptor clusters in eye imaginal discs are two well-studied examples of this form of CCM in *Drosophila *(reviewed in [9, 19]). It has been shown that PCP signaling is involved in several types of CCM. Consistently, PCP components appear to regulate cytoskeletal changes, protrusive membrane activity, cell-cell adhesion and the trajectory of migration, which are essential for cell movement and migration [1, 9, 10, 12].

Numerous reviews have been previously published describing the implication of PCP signaling in different developmental processes in flies and vertebrates. In this review, we remind the contribution of *Drosophila* to our understanding of the PCP molecular machinery, but we will mainly focus on describing recent findings about the requirement of PCP signaling in processes involving CCMs in both organisms. Given the central role of PCP signaling in modulation of cell adhesion, motility and movement in diverse developmental morphogenetic contexts, it seems evident that deregulated PCP signaling is implicated in developmental defects and disease [2, 3, 20]. Besides, recent studies also provide new insights into the link between PCP signaling and cancer [12]. Therefore, new discoveries about the contribution of this pathway to CCMs could provide new potential diagnostic and therapeutic targets for these disorders.

## PCP SIGNALING AND COLLECTIVE CELL MOVEMENTS IN *DROSOPHILA*

*Drosophila *has been extensively used to dissect the signaling pathways that govern multiple developmental processes. As mentioned above, this organism has been crucial for deciphering the molecular mechanisms underlying PCP signaling. Several developmental processes that are regulated by the PCP pathway have been described in flies. In this scenario, we will focus on those involving CCM such as ommatidial rotation (OR) in larval eye imaginal discs, border cell migration (BCM) in the oocyte, and embryonic dorsal closure (DC). 

**Fig. (2):**
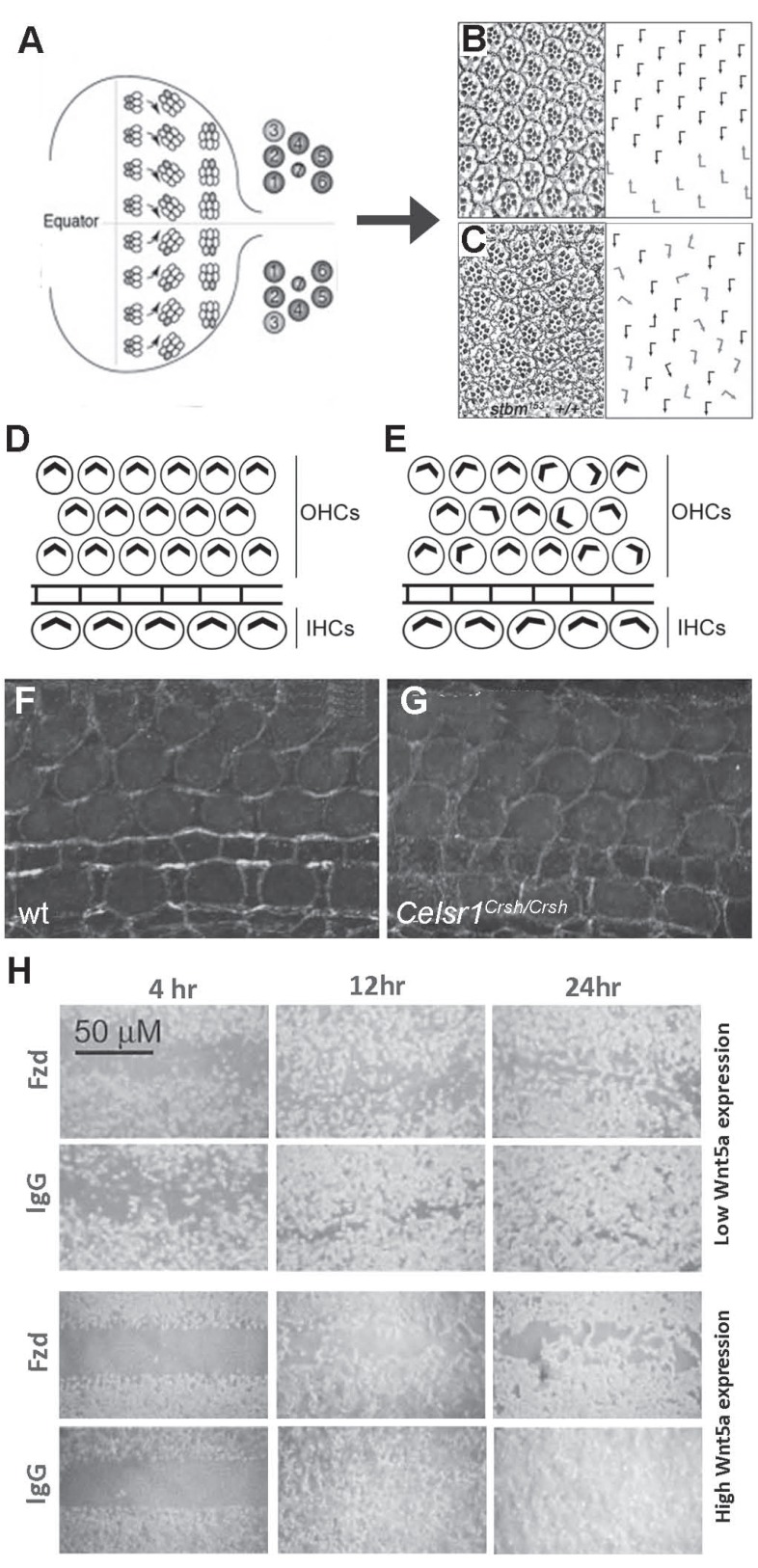
**Effect of PCP signaling disruption in different processes involving CCM at the cellular level**. (**A**) Schematic representation of a third instar *Drosophila* eye imaginal disc showing the equator separating the two eye hemispheres. Anterior is left, dorsal is up. Initially, ommatidial preclusters are symmetrical. PCP signaling leads to determination of R3 and R4 photoreceptors, providing chirality to the ommatidia, then a 90° rotation of clusters towards the equator follows. The mirror-symmetric trapezoidal disposition of the photoreceptors rhabdomeres in the adult is shown in the right. (**B**-**C**) Tangential sections of *Drosophila* adult retinae (left) and schematic representation of the ommatidial orientation (right) in wild type and stbm mutant flies. Arrows represent the angle between each ommatidia and the equator. Reprinted from [157] with permission of Elsevier. (**D**-**E**) Schematics of the hair cells organization in the mammalian organ of Corti in wild type (**D**) and in PCP mutants (**E**). VANGL2 localization in the lumenal surface of the organ of Corti from E18.5 wild type (**F**) and *Celsr1^Crsh/Crsh^* (**G**) mice. A general decrease and a loss of asymmetric localization of VANGL2 are observed in *Celsr1^Crsh/Crsh^* mutants. Reprinted from [84] with permission of Journal of Neuroscience. (**H**) Inhibition of the FZD5 receptor results in an invasion inhibition in scratch assays. WNT5A transfectants, as well as cell lines with high endogenous WNT5A expression were treated with an antibody against the FZD5 receptor in fibronectin-coated chambers. FZD5-treated cells showed a drastic decrease in invasion compared to IgG-treated or untreated cells. Note the still evident scratch in the FZD5-treated cells even after 24 hr. Reprinted from [149] with permission of Elsevier.

During third instar larval development ommatidial preclusters contain several cells that undergo a 90º rotation as a group independently of their stationary neighbors, the interommatidial cells. This process represents a specialized type of CCM known as OR (Fig. **2A-B**). An important advantage for studying this process is that OR defects are easily recognizable and quantifiable in the final eye patterning by tangential sectioning of the fly adult retina. This has allowed to determine that, during OR, signaling from different pathways are integrated to control the achievement of the correct final disposition of ommatidia with respect to the antero-posterior (AP) and dorso-ventral (DV) axes (reviewed in [21]). PCP signaling is involved in OR by influencing different aspects of the process. First, PCP regulates specification of R3/R4 photoreceptors, thus providing asymmetry to the ommatidia. This probably determines the direction of rotation since misrotation is a common phenotype in PCP mutants [22] (Fig. **2C**). Second, modification of cell adhesion properties is essential for OR as it has been evidenced by the opposing effects of E-cadherin (E-cad) and N-cadherin (N-cad) in the process [23]: while E-cad promotes rotation, N-cad acts to restrict this movement. Moreover, mutations in the PCP genes *stbm* and* dgo *as well as reduction of the RhoA GTPase (that functions downstream of the PCP core proteins) activity enhanced the OR phenotype produced by overexpression of a dominant negative form of E-cad in the eye and PCP signaling also contributes to regulate E-cad localization [23]. Additionally, it has been recently shown that the Nemo (Nmo) kinase, encoded by the OR-specific gene *nmo*, is involved in E-cad and β–catenin (β–cat) phosphorylation. Since* nmo* interacts genetically with PCP components [24], it provides a link between PCP signaling and the E-cad and β–cat complex function in the OR process. Interestingly, these observations about the relationship between PCP signaling and E-cad resemble some results obtained in the vertebrate cochlea (see below). On the other hand, a genetic interaction between PCP components and the cell adhesion molecules Echinoid (Ed) and Friend of Echinoid (Fred) has been also reported during OR [25]. Indeed localization of the core PCP protein Fmi is regulated by Ed endocytosis in this context [26]. These results reveal an important role for adhesion molecules in the regulation of PCP signaling during OR. Third, PCP regulates cytoskeleton reorganization via the *Drosophila* Rho kinase (Drok) [27], which is responsible of Myosin II (MyoII) activity regulation. Importantly, the function of Drok and MyoII downstream of the core PCP module has been subsequently shown to be conserved during a process known as convergent extension in mouse, *Xenopus*, and zebrafish [28-31].

During *Drosophila *oogenesis a small group of cells delaminate from a simple epithelium and remain tightly associated as they invade the germline tissue, migrating collectively and in a directed manner in between the giant nurse cells to reach the oocyte [19]. Adhesion between migrating cells and their substrate depends on E-cad [32]. This process is known as border cell migration (BCM) and can be visualized by specific staining of such cells in dissected egg chambers. Interestingly, the velocity of BCM can be determined by comparing to other migrating cells in the egg chamber like the outer follicle cells. It has been shown that PCP signaling is required in the border cells for cluster migration to occur efficiently because knockdown or overexpression of *fz*, *stbm *or *dsh *produces a delay in BCM. This delay is probably due to abnormal cytoskeletal dynamics, since PCP signaling disruption causes abnormalities in actin-rich processes on the cell surface [33]. As it happens in OR, Rho GTPase activity plays an important role in PCP regulation during BCM. Fz localizes to the migratory edges of the border cells, both prior to and during migration [33]. Interestingly BCM is enhanced by PCP activity in the non-migratory epithelial polar follicle cells thus suggesting that PCP can mediate communication between motile and non-motile cells [33].

Another morphogenetic process that requires CCMs and PCP signaling is dorsal closure (DC), which occurs during *Drosophila* embryogenesis. During this process, the lateral epidermal sheets move dorsally to close a hole in the dorsal part of the embryo covered by the amnioserosa [34]. To achieve this, epidermal cells elongate in the DV axis due to the formation of an actomyosin cable at their dorsal-most edge (or leading edge, LE). Actin dynamics is preceded by planar polarization of the dorsal-most epidermal cells (DMC), which is reflected by the redistribution of several cell surface-associated proteins in the plane of the epithelium such as Disc large (Dlg), Fasciclin III (FasIII) and Fmi [35]. Polarization of the DMCs is necessary for them to move in a defined direction. Then, LE cells display defects in polarization in *dsh* mutant embryos, which fail to close properly. Although these results suggest that PCP establishment is necessary for DC, PCP signaling propagation has not been described during this process [36]. Nevertheless, the study of epithelial sheet movements during DC has provided many insights into the regulation of sheet migration. Indeed, DC has been used as a model to study the molecular and cellular mechanisms underlying other epithelial processes such as wound healing [17].

## PCP SIGNALING AND COLLECTIVE CELL MOVEMENTS IN VERTEBRATES

**Fig. (3):**
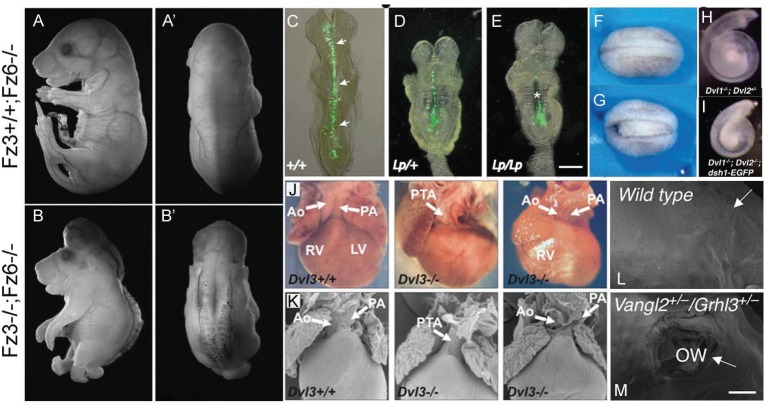
**Mutations in PCP components cause defects in numerous processes involving CCMs in vertebrates.** (**A-B**’) External morphology of *Fzd3*+/+;*Fzd6*-/- (**A**, **A**’) and *Fzd3*-/-;*Fzd6*-/- (**B**, **B**’) mice at E18 in a lateral view (**A**, **B**) and dorsal view (**A**’, **B**’). Note that *Fzd3*-/- ;*Fzd6*-/- mice present a fully open neural tube. Reprinted from [56] with permission of Journal of Neuroscience. (**C-E**) Dorsal views of 5-7 somite stage mouse embryos with a GFP marker of the midline neural plate (arrows). *Lp*/+ (**D**) and *Lp/Lp* (**E**) embryos exhibit limited midline extension, as it can be observed by the GFP labeling. Reprinted from [31] with permission of Development. (**F-G**) Dorsal view of *Xenopus* stage 22 embryos. Control embryos show a completely closed neural tube at this stage (**F**) while dvl2 mutant embryos show neural tube closure defects (**G**). Reprinted from [158] with permission of Development. (**H-I**) Pictures of mouse embryo cochleae. Note that they are shorter and wider in *Dvl1*-/-; *Dvl2*-/- (**I**) than in *Dvl1*-/-; *Dvl2*+/- embryos (**H**). Reprinted from [79] with permission of Development. (**J-K**) Whole mounts (**J**) and scanning electron microscopy images (SEM) (**K**) of *Dvl3*+/+ (left panel) and *Dvl3*-/- hearts of mice at P0 stage that displayed persistent truncus arteriosus (PTA, middle panel) and double outlet right ventricle (DORV, right panel). Ao, aorta; RV, right ventricle; LV, left ventricle. Reprinted from [80]. (**L-M**) SEM of a wound 24h after a hind limb amputation in E16.5 mouse embryos. *Vangl2*+/- ;*Grhl3*+/- mutants (**L**) fail to close the wound while it is completely closed in wild type embryos (**M**). Arrows indicate the boundary of the original wound. OW, open wound. Reprinted from [119] with permission of Elsevier.

In the last years, numerous studies have evidenced the importance of CCMs during different developmental stages in vertebrates. Interestingly, the PCP pathway has been shown to be essential in many of them. Indeed, its disruption leads to the incorrect execution of several developmental processes that involve coordinated cell motility and CCMs during embryogenesis and organogenesis. Moreover, it has been also shown that PCP signaling is linked to processes such as wound repair, and cancer invasion and metastasis in adults because mutations in PCP components cause phenotypes related to them (Table **1**). In this section, we will describe different CCMs that are regulated by PCP components in vertebrates. Examples of phenotypes associated with mutations in PCP components in those processes are shown in Figs. **2** and **3**.

### PCP Signaling in Embryogenesis

Body axis elongation and neural tube closure are two essential developmental processes that occur during embryogenesis. Both involve cell movements and take place through the convergent extension (CE) mechanism, which has been shown to be controlled by the PCP pathway [37]. CE is understood as the narrowing and lengthening of a group of cells. In this complex process, cells elongate mediolaterally and produce polarized cellular protrusions that enable them to move directionally and to intercalate with other neighboring cells [37-39]. This change in shape and movement results in convergence of the cells towards the midline and extension of the tissue along the AP axis. As a consequence, the body axis elongates and the neural tube closes. Then alterations of the CE process lead to defects in both the general body plan and the formation of the neural tube. Other examples of coordinated cell movements regulated by PCP signaling can be found during embryogenesis such as those occurring during eyelid closure, yolk sac formation, and mouse ventral endoderm migration (see below).

Early failure of neural tube closure during embryonic development gives rise to congenital malformations of the central nervous system known as neural tube defects (NTDs) [40]. These defects can affect both the cranial and/or spinal regions of the developing spinal cord [40], and manifest most commonly as anencephaly and/or spina bifida. Neurulation is conserved between mammalian species [41]; therefore, animal models have been instrumental in deciphering the complex molecular mechanisms underlying NTDs [42] and have demonstrated the essential role of the PCP signaling pathway in this process. The mouse mutant *loop-tail* (*Lp*) was the first model that implicated a PCP core gene in the pathogenesis of NTDs [43, 44]. *Lp* mice carry mutations in the vang-like 2 (*Vangl2*) gene [43, 44] and homozygous mutant embryos manifested craniorachischisis, a severe form of NTD characterized by failure in neural tube formation along the entire body axis [45]. Additionally, several studies in vertebrate models have analyzed the effect of mutations in other PCP signaling components like the *fz* homologs *Fzd3 and Fzd6, *the *fmi *homolog* Celsr1, *the* dsh *homologs* Dvl1, Dvl2, Dvl3, *or the* pk *homolog* Prickle1*. These mutants present shorter and wider neural plates than wild-type animals and display craniorachischisis, thus confirming the role of PCP signaling during neural tube closure in vertebrates (reviewed in [46]). Moreover, since CE also affects the final body plan, these mutants displayed a shortened AP axis with concomitant expansion of the mediolateral axis (Fig. **3A-G**). It is noteworthy that screens in patients or fetuses with NTDs in humans have identified missense mutations in *VANGL2, VANGL1, PRICKLE1, CELSR1 *and* FZD6 *[47-52]. Some of these studies have analyzed the functional consequences of these mutations. For example, mutations in *VANGL1 *have been shown to affect in some cases the physical interaction of VANGL1 with the DVL proteins, which is central in PCP signaling [51-53], thus indicating that *VANGL1* is a risk factor in human NTDs. Moreover, subcellular localization experiments revealed that specific mutations in *CELSR1* produced a dramatic reduction of the protein localization to the membrane, a feature that characterizes wild type proteins and is known to be required for PCP pathway function [50]. It is interesting to mention that CCMs that occur during eyelid formation are similar to those occurring in neural tube closure, being this process also controlled by PCP signaling [54-56].

Another critical step during mammalian embryogenesis is yolk sac formation since it provides nutrients and gas exchange to the embryo prior to establishment of the placenta. Without proper yolk sac formation, the embryo either dies or its growth is stunted. Parietal endoderm (PE) migration along the inner surface of the trophectoderm is essential for parietal yolk sac formation. This migration occurs in a manner reminiscent of CE. F9 teratocarcinoma cells provide a convenient *in vitro* model system to study the migration of PE cells [57]. Interestingly, data obtained using F9 cells suggested that the PCP pathway, acting via RHOA/ROCK, regulates oriented cell migration of PE [58], thus involving PCP signaling from the first steps of mammalian embryogenesis in extraembryonic tissues. Finally, recent results show that the formation of multi-cellular rosettes in the mouse ventral endoderm during embryogenesis is also dependent on PCP signaling [59]. Computational simulations showed that the formation of these rosettes is essential for the stereotypic migration of the anterior ventral endoderm, a specialized group of cells responsible for specifying the embryonic anterior pattern [59].

One of the hallmarks of PCP signaling is the asymmetric arrangement of PCP core components with respect to the global axis of the epithelium, originally described in* Drosophila* (see Introduction). This distribution allows cell polarity establishment within the plane of the epithelium, which promotes the rearrangement of cytoskeletal components and translates in directed cell movement like CE. Although subcellular localization of PCP core components has been difficult to determine during CE, studies in animal models have enabled to obtain some data. The asymmetric distribution of PRICKLE1 in the neural plate, determined by inmunohistochemical analyses, has been shown to be essential for neural tube closure in mice [60]. In addition, the use of fluorescent fusion proteins has allowed to determine that Pk localizes at the anterior cell edge during dorsal mesoderm CE movements in zebrafish, whereas Dsh is enriched posteriorly [61]. Similar analyses showed Fz/PCP-dependent Pk localization to the membrane on the anterior side of cells in the notochord and neuroectoderm during zebrafish neurulation [62]. The asymmetric localization of Pk and Dsh observed in zebrafish gastrula is similar to the situation described in flies, suggesting that PCP signaling defines distinct anterior and posterior cell properties to drive cell intercalations during CE. In addition, the importance of misshapen-like kinase 1 (mink1)-dependent pk phosphorylation and rab5-dependent endosomal trafficking for its plasma membrane accumulation and for vangl-pk complex establishment and function during CE has been recently reported in *Xenopus* embryos [63]. Interestingly a genetic interaction between *pk* and *misshapen (msn),* the *Drosophila* ortholog of *mink1*, has been reported in the eye [63] where an endocytic mechanism is also important for regulation of Fmi levels in cell membranes during OR. There is also evidence that the PCP pathway is involved in the localized assembly of extracellular matrix (ECM) and in cell adhesion in vertebrates. Cell-cell/cell–ECM interactions are in turn necessary for the oriented, polarized cell movements required for proper CE and other CCMs (reviewed in [64, 65]). Regarding this, it has been demonstrated that *Vangl2* affects the cytoskeleton and cell adhesion in mouse embryonic neural plate and/or tube. Moreover, the PCP effector RAC1 GTPase plays a key role in this process, so that both a defective and excessive recruitment of RAC1 by VANGL2 produce cytoskeletal abnormalities and impaired adhesion, as it has been shown in cell aggregation and scratch assays using HEK293T, MDCK and C17.2 neural stem cells (65). Thus the correct function of PCP signaling via regulation of VANGL2 levels and its interaction with RAC1 is central for appropriate cell adhesion and neural tube development [66]. Moreover, data obtained in studies of *Xenopus* gastrulation using morpholinos to knockdown genes of interest suggest that localization of the cytoskeleton-related proteins septin 2 and 7 is regulated by the PCP protein fritz, being a crucial control point for CCM [67] and thus linking PCP signaling to cytoskeleton organization via septin proteins during the CE process. Regarding regulation of PCP signaling during CE, it has been shown that several genes can affect PCP signaling at different steps, from regulation of gene transcription to control of protein localization [60, 63, 68-74]. As an example, we already mentioned that mink regulates pk trafficking in *Xenopus* (see above). Moreover, mice mutant for SMAD ubiquitination regulatory factor 1 (*Smurf1*) and *Smurf2* display defects that include a failure in neural tube closure as well as CE defects in the cochlea (see following section). Smurfs have been also shown to participate in PRICKLE1 targeting for ubiquitin-mediated degradation, thus uncovering an unexpected role for SMURF E3 ubiquitin-ligases in controlling PRICKLE1 asymmetrical distribution and hence the dynamic use of the PCP pathway in a local manner during CE [60].

### PCP Signaling in Organogenesis

Although CE was originally observed during gastrulation and neurulation, there is increasing evidence that this mechanism and other types of CCMs regulated by PCP signaling are required for correct formation of vertebrate tissues and organs such as the cochlea and the heart, as well as for NC cells migration.

A process analogous to CE occurs during the development of the organ of Corti, a specialized sensory epithelium in the mammalian cochlea that converts sound-generated pressure waves into neural signals. Sensory cells responsible for this conversion are polarized from the inner to the outer edge and this polarization becomes evident by the localization of modified microvilli, referred to as stereocilia, on their apical surfaces. Two types of hair cells, inner hair cells (IHCs) and outer hair cells (OHCs) are arranged in ordered rows along the length of the cochlear spiral. IHCs and OHCs are morphologically and physiologically distinct from one another (Fig. **2D**). IHCs are the predominant cells that respond to sounds, while OHCs primarily modulate the response of the organ of Corti to a particular sound (reviewed in [75]). Thus, CE defects in this epithelium correlate with sensory hair cells polarization defects and deafness (Fig. **2E**), as stereociliary bundles are only sensitive to vibrations in their single plane of polarization. In addition, the organ of Corti also contains distinct types of nonsensory cells, named supporting cells. CE-like movements occur after sensorineural precursors have exited mitosis, therefore this process is independent from oriented cell division. Morphological analysis as well as immunostaining in the inner ear have demonstrated that CE-related shape changes in the cochlea fail to occur in *Vangl2,* *Dvl3, Fat4 and Wnt5a *mutants, and in *Dvl1;Dvl2* and* Fz3;Fz6* double-mutants (Fig. **3H-I**) [76-81]. Moreover the PCP regulators Scribble1 (SCRB1) and Protein tyrosine kinase 7 (PTK7) also play a role in CE processes in the cochlea [77, 82]. The characteristic asymmetry in PCP components localization is also evident in vertebrate auditory organs. In the chicken inner ear, CELSR1 localizes asymmetrically in both hair cells and supporting cells in the sensory epithelium of the basilar papilla, the avian analogous to the organ of Corti [83]. In the mouse organ of Corti, DVL2-EGFP fusion protein localizes asymmetrically at the outer cell surface where the actin-rich stereocillia form, and this localization is lost in the *Vangl2* mutant *Lp* [78, 79]. VANGL2, on the other hand, localizes to the inner side of sensory cells in wild type condition and this localization is dependent on CELSR1 (Fig. **2F-G**) [84]. This localization reminds that observed for the *Drosophila* ortholog proteins in wing cells. In contrast, FZD3 and FZD6 co-localize asymmetrically with VANGL2, and this localization is also lost in the *Vangl2* mutant [56, 84]. In this case, FZD proteins present a different localization to that observed in *Drosophila, *implying that a fundamental difference in PCP establishment exists between flies and mammals. Since DVL localization depends on FZD recruitment, these observations leave unresolved why DVL localizes to the outer cell surface in cochlear cells [9]. In addition PCP defects in *Celsr1^Crsh/Crsh^* mutants, in which Vangl2 asymmetry is lost, are less severe than those in null *Vangl^Lp/Lp^* mutants suggesting that membrane-localized Vangl2 retains some ability to generate a polarizing signal, even in the absence of asymmetric localization [84]. Examination of cellular morphology during cochlear extension revealed that cellular contacts and geometry change drastically, and analysis of the expression of adherens junctions components showed that dynamic expression of N-CAD and E-CAD demarcates sharp boundaries in this process [85]. The conditional knockout of the p120-catenin gene (*p120^CKO^*), which encodes a component of the adherens junctions, leads to reduction of E-CAD and N-CAD levels. Moreover *p120^CKO/CKO^* animals present characteristic cochlear CE defects (50% penetrance) that are enhanced by addition of a single *Vangl2 *loss-of-function allele (complete penetrance). Loss-of-function *Vangl2* mutants, also present altered dynamic distribution of N-CAD and E-CAD in the cochlea [85]. These data provide additional evidence of the role of PCP in the regulation of adherens junctions in CCMs, and support a role for *p120-cat* in PCP signaling during CE [85]. These results are in contrast with the observation that unidentified signals independent of PCP genes control the polar distribution of E-cad during CE movements in *Drosophila*, such as those occurring in germ band extension during embryogenesis [85, 86]. However it is noteworthy that in other *Drosophila* CCM processes like OR, distribution of E-Cad is in fact regulated by the PCP pathway [23].

The NC is a multipotent cell population specified at the interface between the neural and non-neural ectoderms [87]. After induction, NC cells separate from their surrounding tissues during a delamination phase and conduct an extensive migratory behavior. As a result, they colonize nearly all tissues and organs of the embryo [88] where they give rise to a wide range of derivatives [89]. Most of these cells migrate collectively as interactions between cells directly influence cell directionality and are essential for the interpretation of external cues [90]. One of the key factors that control directional migration of NC cells is the PCP signaling pathway. Functional inhibition of different PCP pathway components and potential modulators block the migration of cranial NC cells (reviewed in [18]). These migration defects have been related to the direction in which cell protrusions are formed [18], so that a key function of PCP signaling is to restrict lamellipodial protrusions to the NC cells leading edge in a way that involves the small GTPases RhoA and Rac [90, 91]. It has been also shown that the proteoglycan Syndecan-4 and PCP signaling work in a coordinated manner to control the directionality of NC cells migration both *in vitro* and *in vivo*, by regulating cell polarity and the cytoskeletal machinery that controls the formation of cell protrusions through regulation of RhoA and Rac1 activation, as it has been evidenced by FRET experiments [91]. It has been also proposed that a PCP-like pathway, in which the Wnt11r ligand binds to the Muscle-specific receptor kinase (MuSK) to initiate a Dsh-dependent signaling cascade, also participates in the restriction of NC cells migration to specific segmental paths in zebrafish [92].

Congenital heart disease, the most common congenital disorder in humans, occurs in approximately 1% of live births [93]. In particular, those that involve the outflow tract like defects of the transposition of the great arteries (TGA), double outlet right ventricle (DORV), and persistent truncus arteriosus (PTA), where a single outflow tract vessel is observed in place of the normal aorta and pulmonary artery, are especially prevalent [43]. Normal development of the cardiac outflow tract requires migration of secondary heart field (SHF) cells from the pharyngeal mesoderm and their addition dorsal to the primary heart tube [94, 95] to contribute to the myocardium of the outflow tract [96]. This migration takes place through a process similar to CE. Then addition of cardiac neural crest (CNC), mesenchyme from the crest of the neural folds, is required to septate the single vessel to form the aorta and pulmonary artery [97]. Defects in these cell migrations cause the above mentioned phenotypes. It has been reported that *Lp* homozygous mutant mice display DORV abnormalities due to a disruption in polarized migration of myocardial cells to the outflow tract septum [98, 99]. These abnormalities, together with TGA and PTA, have been observed in *Wnt11* [100], *Wnt5a* [101], *Dvl2* [102] and *Dvl3* mutant mice (Fig. **3J-K**) [80]. In addition, mutations in PCP components in zebrafish also result in CE heart defects (reviewed in [103, 104]). Since PCP has an important role from early steps in embryogenesis, cardiovascular abnormalities could be considered as a secondary consequence of the defects in embryonic patterning caused by PCP signaling failure. However, it is noteworthy that not all PCP mutants with heart defects present NTDs [80, 105]. These results reveal an important role for PCP in heart morphogenesis.

### PCP Signaling in Epithelial Repair

Wound healing (WH) is another process that requires coordinated epithelial sheet migration in order to repair different tissues and organs after injury [106-108]. In skin wounds, closure occurs by coordinated and polarized cell migration during the proliferative phase, where epithelialization, proliferation, and angiogenesis are involved (reviewed in [17]). During WH, keratinocytes maintain close contacts that are mediated by E-CAD and desmosomal proteins and exhibit PCP. This allows a coordinated and directional movement as a monolayer sheet in the plane of the epithelium using integrins and signals from fibrobrasts [1, 109-112]. This process is integrated and regulated by the actin cytoskeleton through the small GTPases of the Rho subfamily by two distinct mechanisms. In embryonic wounds, they control the formation of actomyosin cables, which are organized into a purse string at the wound margins and ultimately pull them together. In adult wounds, however, Rho small GTPases control the formation of actin-ring structures and membrane protrusions (lamellipodia/filopodia) towards the direction of migration of the wound edges [113-118]. 

Although several evidences already suggested that PCP signaling could be linked to WH, a recent study in mice has demonstrated for the first time that this pathway is required for actin polymerization and polarization of subcellular organelles at the LE of free cell sheets [119]. The authors identified the epidermal transcription factor Grainy head-like 3 (GRHL3) as important contributor to PCP propagation and established a novel mechanism by which the precise spatial-temporal expression of small GTPases is required for cell migration and polarity [119]. Previous studies already showed that *Grhl3 *mutant mice displayed defective skin barrier function and deficient wound repair, but they also showed NTDs and failure of eyelid closure [55, 120, 121]. Interestingly, GRHL3 is the homolog of the *Drosophila* Grh protein, which is essential for the Fz/PCP pathway and is involved in epithelial barrier formation, WH, regulation of wing hair formation, and ommatidial orientation in flies [122, 123]. It has been demonstrated that *Grhl3* expression is markedly up-regulated in mammalian cells at the wound margins, which is required to establish directional polarity at the wound LE. This up-regulation is achieved through direct transcriptional activation of the PCP effector gene *RhoGEF19* by GRHL3 [119]. *RhoGEF19* encodes the ortholog of xwgef, a guanine-nucleotide exchange factor (GEF) required for rhoa activation and involved in PCP signaling in *Xenopus* [124]. Moreover, it was shown that knockdown of *Grhl3 *or *RhoGEF19* in keratinocytes induced defects in actin polymerization, cytoskeletal arrangements, and directional migration during WH [119]. Although the nature of upstream factors that could regulate *Grhl3* is still unknown, the authors suggested that FGF signaling activation in response to wounding could induce *Grhl3* expression, resulting in *RhoGEF19 *up-regulation and RHOA activation and leading to cellular polarity and coordinated directional cell migration [119]. These authors also described that other PCP signaling components, including the FZD6 receptor and its effectors VANGL2 and CELSR1, are required for cell patterning across the surface of the skin during the epidermal wound repair in mice [119]. In addition, PCP signaling has been linked to endothelial cell proliferation and angiogenesis, suggesting putative new functions of PCP pathway in the WH process [125, 126]. The study of the molecular and cellular mechanisms involved in WH will allow to understand epidermal pathologies such as chronic WH defects (ulceration) or overhealing (hypertrophic, contracture scars and other fibroproliferative disorders). Regarding this, it remains to be determined whether junctional or cytoskeletal components in basal cells affect signaling via PCP proteins to regulate epidermal WH or morphogenesis. Studies in invertebrate and vertebrate models combined with *in vitro* scratch WH assays and high-throughput imaging analyses can help to identify new PCP components involved in wound repair and other related physiological process [127]. 

### PCP Signaling in Cancer

As mentioned above, PCP establishment is critical during embryonic development, a process which shares many similarities with cancer development, but it is also required during WH. Regarding this, some authors support the theory that cancer is a manifestation of development gone awry [128, 129]. In fact, several signaling pathways involved in embryogenesis are deregulated in tumourigenesis [12]. Alternatively, other authors hypothesize that cancer could arise from wounds that do not heal or from overhealing wounds, which is supported by the fact that WH and cancer share several features such as loss of cell-cell adhesion and cell polarity [128, 130, 131]. Taken together, these ideas suggest that PCP signaling could be important in tumour formation. Consistently, it has been recently described that *Grhl3* is implicated in the prevention of skin cancer in mice [132]. Aberrant activation of WNT/PCP signaling pathway in human cancer leads to more malignant phenotypes, such as abnormal tissue polarity, invasion, and metastasis. However, the precise role of PCP signaling in these processes remains still controversial. Several studies have shown that PCP signaling could play opposite roles in tumourigenesis, with early tumour suppressive effects through growth inhibition maintenance of cell-cell comunication-signaling and cell homesotasis [133-138], but later promoting cancer progression through regulation of tumour invasion, metastasis, and angiogenesis [139, 140]. Accordingly, several components of the PCP signaling pathway present this biphasic role in mammals such as Fat, Dchs, Paraxial protocadherin (PAPC) and its human ortholog protocadherin-8 (PCDH8), Dvl and Wnt5a [135, 137, 138, 141-143]. It has been shown that Fat, Dchs, and PAPC can suppress tumour progression, although PAPC might also enhance tumour development. For example inactivation of PCDH8 in breast through either genetic alteration or epigenetic silencing of expression, promoting oncogenesis by the repression of mitogenic signaling and disrupting cell–cell communication dedicated to tissue organization and homeostasis [138]. Moreover Dvl family proteins are overexpressed in nonsmall cell lung cancer and promoting lung cancer cell invasion by different ways. *Dvl-1* overexpression enhanced the Tcf-dependent transcriptional activity and β-cat expression significantly (canonical Wnt pathway). However, *Dvl-3* had little effect on the Tcf-dependent transcriptional activity and β-cat expression, which was accompanied by p38 and JNK phosphorylation (noncanonical Wnt pathway) [140]. Besides, Wnt5a acts as an oncogene or tumour suppressor gene in a context-dependent manner [135, 137, 138, 141-143]. Several evidences obtained from studies in *Drosophila* support the idea that disruption of cell polarity mechanisms combined with an increase in cell growth plays a causal role in tumour initiation and progression. Indeed, loss of proteins that interact with the PCP pathway in determining both planar and apico-basal polarity, such as Disc large (Dlg), Lethal giant larvae (Lgl) and Scrib, causes hyperproliferation, loss of apical-basal polarity and abnormal cell shapes (reviewed in [131]). Moreover, changes in activity, expression and/or localization of core cell polarity proteins have been described in vertebrate tumours, and there are also evidences that basic cell polarity mechanisms are often targeted by oncogenic signaling pathways (reviewed in [134]). Several studies in mammals also suggest that PCP signaling interfere with other signaling pathways such as ERK/MAPK or RAC1/JNK to influence proliferation [12, 131, 144, 145]. In addition, non-canonical Wnt signaling has been considered to have a tumour suppressor role, since it maintains quiescence of stem cells and inhibits canonical Wnt signaling (reviewed in [3]). Finally, PCP signaling appears to be also involved in tumour metastasis, in which cancer cells of several types develop the ability to move collectively and invade new tissues and form secondary tumours [146, 147]. Although the mechanisms involved in tumour invasion and metastasis are still not well understood, several studies have shown the role of PCP signaling in cell adhesion, motility, and coordinated movements that are critical in this process. Indeed several PCP components and/or modulators have been shown to promote metastasis in different human cancer types, such as WNT5A in melanoma, gastric cancer, and breast cancer by activating Rac and JNK [148-150], FZD7 in hepatocellular carcinoma and colon cancer, FZD10 in synovial sarcoma [151-154] or VANGL2, which promotes cellular migration and ECM invasion of fibrosarcoma tumour cells [12, 128, 155] (Fig. **2H**). Interestingly, it has been shown that silencing of Vangl1 suppresses colon cancer metastasis in mice, thus confirming its metastasis-promoting function [156]. Taken together, these results indicate that PCP signaling plays important roles in tumourigenesis (reviewed in [12]). Therefore, some PCP components and modulators have the potential to be used as biomarkers and powerful targets for cancer therapy.

## CONCLUSIONS AND PERSPECTIVES

PCP signaling has emerged as an important regulator of directed and CCMs occurring during fly and vertebrate development, but it is also linked to processes such as wound repair, and cancer invasion and metastasis. Due to the remarkable conservation of the PCP molecular machinery, further genetic analyses in *Drosophila* will undoubtedly help to discover new mechanisms underlying PCP establishment during these processes. Of additional interest will be to identify new upstream regulators as well as effectors of PCP signaling, and to determine whether crosstalk with other signaling pathways is important for its function in different contexts. However, since recent studies have shown that several mechanistic aspects of PCP as well as components of PCP signaling are vertebrate specific, much work is still needed in this direction. These studies will be also relevant for several reasons. As mentioned above many developmental defects associated with PCP have been described in vertebrates. However, it still remains unknown whether other human diseases in which CCMs are defective could arise from incorrect PCP establishment. In this direction, screens for mutations in PCP components in patients with NTDs have identified mutations that could be responsible for these defects. Similar experiments in other CCM-related disorders would probably provide additional data; therefore analyses of the potential functional effect of these mutations would be of special interest. Since CCM processes are difficult to study in developing organisms, the application of new techniques such as F9 cells assays or computational simulations as well as the analysis of protein interactions and/or PCP pathway activity would be also required to shed light into the molecular and mechanistic events underlying those processes. Interestingly, recent studies have also demonstrated that PCP signaling plays important roles in WH and tumourigenesis. Therefore, new discoveries about the contribution of this pathway to CCMs either during morphogenesis or in pathological conditions could help to develop new potential diagnostic and therapeutic approaches for human diseases and cancer.

## Figures and Tables

**Table 1.** Summary of PCP Genes Whose Mutations Produce CCM Related Defects in Vertebrates

CCM Process	PCP Genes Involved	Organism	References
Neural tube closure	*Vangl1, Vangl2, Fzd3, Fzd6, Celsr1, Dvl1-3, Prickle1 *	Human, mouse, Xenopus	[43-52], [158]
Body axis elongation	*Vangl2, Fzd3, Fzd6, Celsr1, Dvl1-3, Prickle1 *	Mouse	[43-46]
Parietal endoderm Migration	*RhoA/ROCK *	Murine F9 cells	[58]
Ventral endoderm Migration	*Celsr1*	Mouse	[59]
Inner ear development	*Vangl2, Dvl1-3, Fat4, Wnt5a*, *Fzd3, Fzd6, Scrb1*, *Ptk7 *	Mouse	[76-82]
Neural crest cell Migration	*Dvl, Wnt11, tri * (*strabismus*), *RhoA*, *Rac *	Xenopus, zebrafish	[18], [91], [159]
Heart development	*Wnt11, Wnt5a*, *Dvl2*, *Dvl3*	Mouse	[100-102]
Wound repair	*Grhl3, Vangl2, Celsr1, PTK7, Scrb1 *	Mouse	[119-121], [132]
Cancer	*Wnt1, 5a, 11, Fzd7-10, Dvl1, 3, Vangl1, Celsr, Prickle *	Human, mouse	Reviewed in [12], [140], [149], [151], [154], [156]
